# Protein profiling of water and alkali soluble cottonseed protein isolates

**DOI:** 10.1038/s41598-018-27671-z

**Published:** 2018-06-18

**Authors:** Zhongqi He, Dunhua Zhang, Heping Cao

**Affiliations:** 1 USDA-ARS, Southern Regional Research Center, New Orleans, Louisiana USA; 2 USDA-ARS, Aquatic Animal Health Research Unit, Auburn, Alabama USA

## Abstract

Currently, there is only limited knowledge on the protein types and structures of the cottonseed proteins. In this work, water-soluble cottonseed proteins (CSPw) and alkali-soluble cottonseed proteins (CSPa) were sequentially extracted from defatted cottonseed meal. Proteins of the two fractions were separated by 4–20% gradient polyacrylamide gel electrophoresis (SDS-PAGE); There were 7 and 12 polypeptide bands on SDS-PAGE of CSPa and CSPw, respectively. These individual bands were then excised from the gel and subjected to mass spectrometric analysis. There were total 70 polypeptides identified from the proteins of the two cottonseed preparations, with molecular weights ranging from 10 to 381 kDa. While many proteins or their fragments were found in multiple bands, 18 proteins appeared only in one SDS-PAGE band (6 in CSPa, 12 in CSPw). Putative functions of these proteins include storage, transcription/translation, synthesis, energy metabolism, antimicrobial activity, and embryogenesis. Among the most abundant are legumin A (58 kDa), legumin B (59 kDa), vicilin C72 (70 kDa), vicilin GC72-A (71 kDa), and vicilin-like antimicrobial peptides (62 kDa). This work enriched the fundamental knowledge on cottonseed protein composition, and would help in better understanding of the functional and physicochemical properties of cottonseed protein and for enhancing its biotechnological utilization.

## Introduction

As a crop of fiber source for textile globally, cotton is produced in more than 80 countries. The most widely cultivated cotton species today are *Gossypium hirsutum* and *G*. *barbadense*^1^. Much of the cotton land area in the US is located in the southern and southeastern regions^2–4^. Although cotton is mainly planted for its fiber, for every 100 kg of lint fiber ginned from cotton, 150 kg of cottonseed is produced^5–7^. The cottonseed mainly contains lipids, proteins, carbohydrate, and minerals^8–12^. The lipid fraction (oil) is mainly used in the food industry^5^, and has the potential for biodiesel production as petitioned to U.S. Environmental Protection Agency Fuels Programs Registration by US National Cottonseed Products Association^13^. The whole cottonseed and defatted cottonseed meal have been frequently used in animal feeds and garden fertilizers^14–16^. Recently, the industrial applications of the functional components of proteins and peptides in the cottonseed meal and its protein isolates are very promising. The potential value-added products of cottonseed protein isolates include but are not limited to bioplastics and films^17,18^, superabsorbent hydrogel^19^, antioxidant peptides/extracts^20,21^, and bio-based wood adhesives^22,23^. Studies^22,24^ have shown the differences in the adhesive performance between cottonseed protein adhesives and widely-studied soy protein-based adhesives, which may be attributed to the difference in protein structures and composition between the two types of oil seeds.

In cotton seed, two major classes of storage proteins are globulins and albumins, which differ in their solubility properties. Both globulins and albumins are synthesized and compartmentalized in protein storage vacuoles during cotton seed maturation. Globulins can be further classified based on sedimentation rate of their aggregated forms into the 7 S vicilins (or α-globulin) and 11/12 S legumins (or β-globulin)^25,26^. Both vicilin and legumin families comprise the major (60–70%) components of cotton seed proteins revealed by the proteomic profiles of mature cotton seeds^27^. There are also some functional proteins in cottonseed. For example, oleosins in cottonseed play dual physiological roles, by acting as protectors for stabilizing the oil bodies in developing and mature seeds and as the recognition signal for lipase binding in germinating seeds^26^. Using a newly developed quality trait loci mapping method^28^, it has been shown that essential amino acid contents in cottonseeds can be improved via environmental manipulations. Therefore, more knowledge on the protein types and their structures is needed for better understanding and utilization of cottonseed proteins. For this purpose, in this work, we isolated and separated cottonseed proteins into water- and alkali-soluble fractions, and analyzed their polypeptide profiles.

## Results and Discussion

### Polypeptide bands of CSPw and CSPa on gradient SDS-PAGE

**Figure 1:**
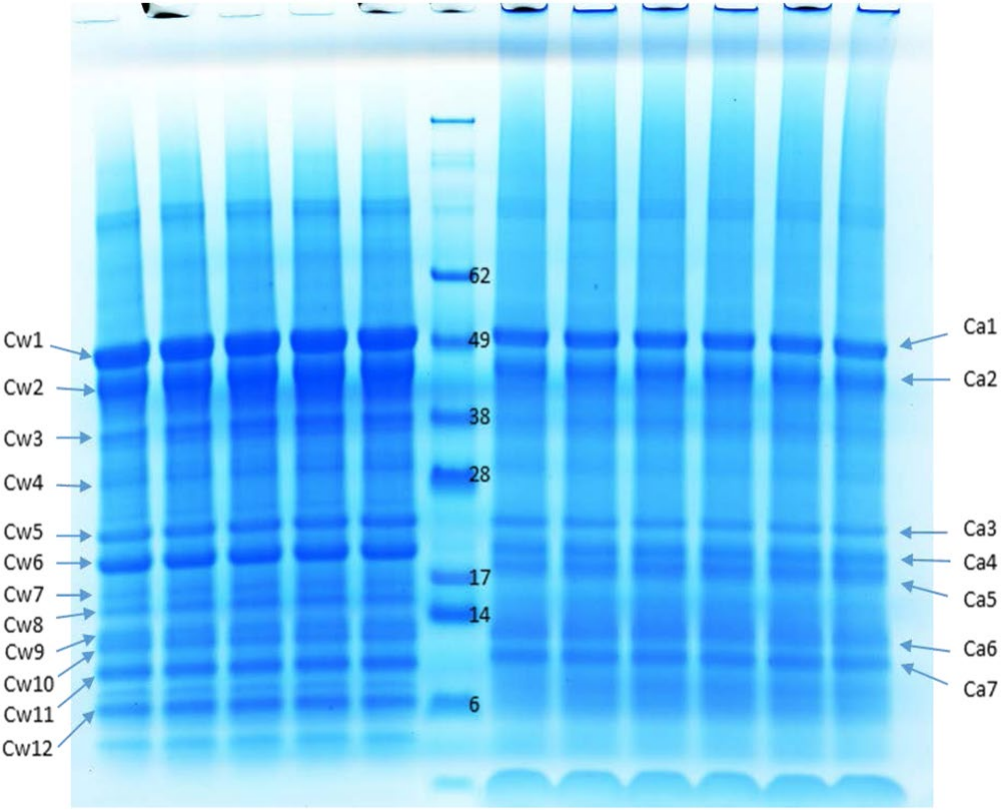
Gradient (4–12%) SDS-PAGE of two-step prepared water- (CSPw, left) and alkali- (CSPa, right) soluble cottonseed protein isolates. Approximately 5 µg of protein were applied to each lane).

Intrinsic fluorescence excitation–emission matrix spectroscopy^29^ has shown CSPw is hydrophilic but CSPa is more hydrophobic. The distinction between the cottonseed protein fractions was also obvious in the polypeptide patterns as shown in the gradient SDS-PAGE image (Fig. 1). Previously, 10 to 13 polypeptide bands were reported in cottonseed protein isolates^30–32^. Separation of the total cottonseed protein into CSPw and CSPa improved the resolution of SDS-PAGE as 12 polypeptide bands of CSPw sample and 7 bands from the CSPa sample could be identified. Whereas the amount of CSPw is about 20% of CSPa in cottonseed meal^32,33^, there were more bands in CSPw sample than in CSPa. Some proteins in CSPw apparently appeared in different molecular weights with more bands below 20 kDa. With such features, in addition to the intact protein polypeptides, some bands of CSPw with smaller molecular mass shown at the SDS-PAGE image might be released fragments of the longer polypeptides during sample treatment.The molecular mass of many proteins identified seemed greater than that in the gel image. This could be due to protein being broken-down (hydrolysis and/or disulfide bond reduction). For example, a recent study^34^ has shown the difference in the SDS-gel patterns of the cottonseed protein products treated by oven-, spray-, and freeze-drying, which was assumed due to the heat-induced protein polypeptide alterations.

### Peptide and protein profiles of CSPw and CSPa

**Figure 2:**
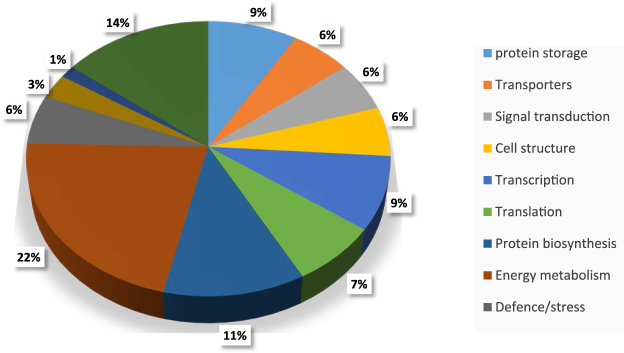
GO term predicted biological functions and percentage of each class of proteins identified in cottonseeds.

A total of 2,319 exclusive unique peptides (with 99% threshold) was revealed from the 19 excised gel samples (Table 1). These peptides were the most matched gene products of *Gossypium arboreum* and/or *G*. *hirsutum* except for a cytosolic phosphoglycerate kinase found in an unspecified *Gossypium* species (Supplemental Fig. 1), which might be attributed to the fact that the genomes of *G*. *arboretum* (37) and *G*. *hirsutum* (38) were sequenced and available for comparison. These unique peptides belong to 70 proteins with molecular weights ranging from 10 to 381 kDa. The samples CSPa and CSPw have 56 and 49 protein species, respectively. There were about 20 proteins identified from these peptides of each alkali-CSPa gel band (Table 1). Whereas there were more gel bands in CSPw sample, these bands contained less protein types (5–19) than CSPa gels. Biological functions of these proteins, predicted by GO term, include protein storage, transporters, signal transduction, cell structure, transcription, translation, protein biosynthesis, protein metabolism, energy metabolism, antimicrobial activity, defense/stress, carbohydrate metabolism, and fatty acid metabolism, with 14% protein species of unknown functions (Fig. 2). Proteins for storage (9%), transcription (9%), biosynthesis (11%) and energy metabolism (22%) accounted for about half of the proteins identified. Identification of the proteins present in the cottonseed would be helpful in gene expression studies of cotton crop. For example, the peptide match analysis found three putative proteins (ATP-dependent RNA helicase DHX36, vacuolar sorting-associated protein 13B, and zinc finger CONSTANS-LIKE 11-like protein), confirmed their gene expression patterns (i.e., true transcription/translation) from cotton genomes. On the other hand, quantitative analysis of the abundance of the polypeptides by total ion current in MS analysis (Supplemental Fig. 2) showed that these functional proteins account only small or even tiny fractions of the whole cottonseed protein, while vicilin- and legumin-related polypeptides overwhelmingly dominate as the major storage proteins.

**Table 1:** Exclusive unique peptide count of CSPw and CSPa.

Identified Proteins (70)	MS	CSPa							CSPw											
	Kda	1	2	3	4	5	6	7	1	2	3	4	5	6	7	8	9	10	11	12
Vicilin GC72-A	71	20	29	29	25	26	21	21	24	28	24	25	25	22	27	24	26	25	22	18
Vicilin C72 G. hirsutum	70	38	28	28	20	29	16	16	39	33	38	29	20	23	32	31	29	30	28	16
Legumin B	59	13	12	14	26	17	15	14	11	15	22	24	21	21	23	22	23	27	20	16
Legumin A	58	13	24	24	35	23	28	34	17	28	29	31	30	38	30	27	30	28	38	23
Vicilin-like antimicrobial peptides 2-1	62	6	9	13	2	3	4	6	8	8	15	6	9	2	6	6	3	4	6	
Vicilin C72 G. arboreum	70	7	2	1	3	3	1	1	7	4	4	1	2	3	2		2	1	1	
ATP synthase subunit beta	60	3	1	2	1	3	1	1												
Elongation factor 1-alpha	49	4	2	2	1		1	2												
Malate dehydrogenase-2C cytoplasmic	36	2		2	1	2	1													
Eukaryotic initiation factor 4A-14	35	1	1	2			1	1												
Glyceraldehyde-3-phosphate dehydrogenase-2C cytosolic	18			1	1	1	2	1												
Late embryogenesis abundant protein	17		2	1	1	1														
ALBINO3-like protein 1, chloroplastic	59				1	2		1												
Desiccation-related PCC3-06	26			3	1	1														
Histone H2B	25			2			1	1												
Low molecular weight heat shock protein	18				1	2		1												
Protein NLP8-like protein	107		1			2														
Phosphoglycerate kinase, cytosolic	42	1	2																	
Fructose-bisphosphate aldolase, cytoplasmic isozyme	39	1	2																	
Proteasome subunit beta type-6-like protein	25			1	2															
60 S ribosomal L23a	18				1	2														
1-acylglycerophosphocholine O-acyltransferase 1	64			2																
Phototropin-1-like protein	61	2																		
V-type proton ATPase subunit B 1	54						2													
Isocitrate dehydrogenase [NADP]	46		3																	
40 S ribosomal S3-3-like protein	26			2																
(3 R)-hydroxymyristoyl-[acyl-carrier-protein] dehydratase	24					2														
Putative vacuolar sorting-associated protein 13B	381								2	1										
HEAT repeat-containing 7 A	187									2	1									
Golgi to ER traffic 4	194																2			
Origin recognition complex subunit 1	104											2								
Exocyst complex component 7	75									2										
ADP,ATP carrier 1, chloroplastic-like protein	69									2										
Vacuolar-sorting receptor 1-like protein	69								2											
Guanylate-binding 5	66												2							
Kinase PVPK-1	65								2											
Rhamnogalacturonate lyase	61																2			
Protein yeeZ	40												2							
Calcineurin subunit B	20								2											
Chaperone DnaJ	13									2										
Serine/threonine-protein kinase SIK3	13								2											
2 S albumin storage protein	16		1	1	1	2		2			1	1				3				1
Late embryogenesis abundant protein D-19	11	1		2	2	2	2	3							1	1	1			
Vicilin-like antimicrobial peptides 2-2	55			4		3						1		1	1		1	1		
Protein lin-54	82	1					1		1	1	2						1			
Putative ATP-dependent RNA helicase DHX36	117	1	2		1								1	1						
Transcription initiation factor TFIID subunit 1-B-like protein	111	1								3			1				1	1		
Heat shock protein 70	71		1	4		4		1				1								
40 S ribosomal S5	23			1	2	1	1							1						
Oleosin 16.4 kDa	16						4	1								1	1	1		
DNA polymerase alpha catalytic subunit-like protein	172				1				1	1		2								
Glucose-6-phosphate isomerase, cytosolic	60			1		1			1	2										
RING finger and CHY zinc finger domain-containing 1	18	1	1							1						2				
Flagellar attachment zone 1	184				1			1									3			
Histone-lysine N-methyltransferase ATX2-like protein	123	2						1	1											
Protein neuralized	99		2				1								1					
Protein argonaute 4-like protein	97							1	2			1								
Replication A 70 kDa DNA-binding subunit	65		2							1		1								
Adenosylhomocysteinase	53		2														1	1		
Peptidyl-prolyl cis-trans isomerase cyp8	37		1					1										2		
Cellular nucleic acid-binding	36							1	2										1	
Mitochondrial 2-oxoglutarate/malate carrier	32		1							2							1			
60 S ribosomal protein L14-2	21					1	2										2			
Zfy1	10					1									2	1				
CBL-interacting serine/threonine-protein kinase 23	172						2								1					
Beta-xylosidase/alpha-L-arabinofuranosidase 2	84					1													2	
Chaperonin CPN60-2, mitochondrial	61			2											1					
Lipid A export ATP-binding/permease MsbA	55					1				2										
Transcription factor BIM2-like protein	36						1		2											
Putative zinc finger CONSTANS-LIKE 11-like protein	29		2						1											
Total unique peptides		118	133	144	130	136	108	112	127	138	136	125	113	112	127	118	129	121	118	74
Total proteins associated		19	24	24	22	26	21	22	19	19	9	13	10	9	12	10	17	11	8	5

Among the 70 protein polypeptides, 4 dominant proteins [i. e., legumin A, legumin B, vicilin C72 (*G*. *hirsutum*), and vicilin GC72-A] appeared in all 19 gel bands. Three additonal proteins appeared in all 7 CSPa bands (vicilin C72, vicilin-like antimicrobial peptides 2–1, and ATP synthase subunit beta). With a little less abundance (Supplemental Fig. 2), Vicilin C72 and vicilin-like antimicrobial peptides 2–1 proteins also appeared in 10 or 11 of 12 gel bands of CSPw (Table 1). These protein should be the major components of cottonseed proteins. Another storage protein (2 S albumin storage protein) was distributed in both, but not all, CSPa and CSPw gel bands, perhaps due to its low fraction of the total seed proteins (Supplemental Fig. 2)^35^. It should also be noted that mature cottonseed albumins are typically cleaved into smaller polypeptides that fall outside of the effective separation range of SDS–PAGE. Thus, there is a possibility that a poor representation of albumin in the present protein profiles due to this technical limitation of SDS–PAGE^27^ as it was reported recently that 2 S albumin transcripts were more abundant compared to either legumins or vicillins^36^.

These funtional proteins were less widely distributed in the SDS-PAGE gel bands. 20 proteins were found in CSPa fraction only, and 14 proteins were found in CSPw only. There were 18 proteins that appeared only in one SDS-PAGE band (6 in CSPa lanes, 12 in CSPw lanes).

### Features of the peptides that appeared in multiple SDS-PAGE bands and multiple proteins in one band

As there were 18 proteins that appeared on one single band (e.g., band 1 in CSPa) and the same protein that appeared in multiple bands (e.g., vivilin GC72-A in all 19 bands) (Table 1), one would ask for explanations on the features (or causes) of those peptide fragments (or associated proteins) which appeared in multiple SDS-PAGE bands. There seemed to be three mechanisms to explain the types of observations: 1) contamination from gel and separation column, 2) degradation in protein isolation and post treatments, and 3) indigenous isoforms (i. e., genetically controlled protein modification).

**Figure 3:**
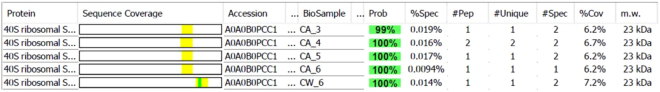
Sequence coverage of 40 S ribonsomal S5 (A0A0B0PCC1, G. arboreum) by peptide fragments in CSPw and CSPa.

**Figure 4:**
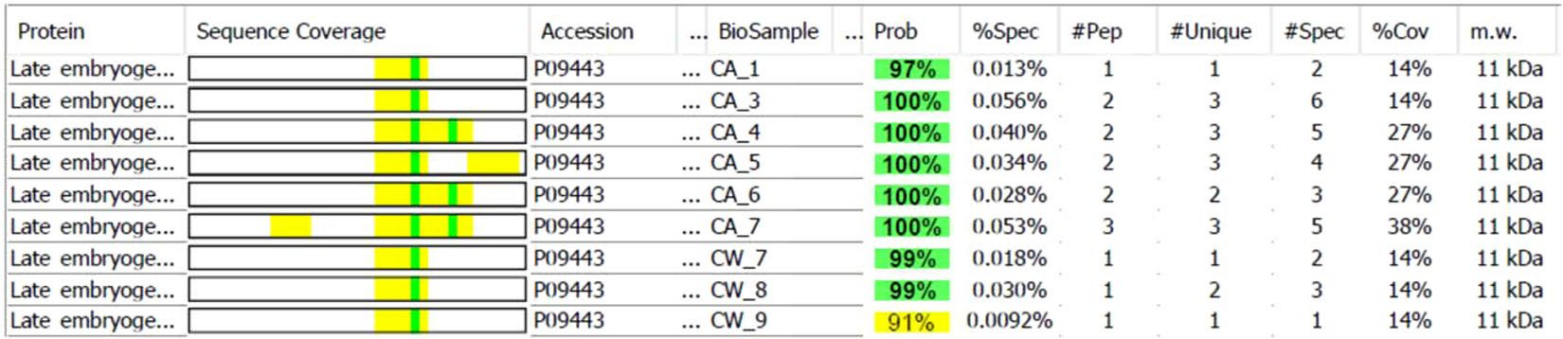
Sequence coverage of late embryogenesis abundant protein D-19 (P09943, G. hirsutum) by peptide fragments in CSPw and CSPa.

The cross contamination may occur during protein gel and column separations. We assumed, however; that the simple contamination would not have altered the protein structures so that the peptide fragments which appeared in multiple SDS-PAGE bands would be identical. For example, ALBINO3-like protein 1, chloroplastic, was identified in Ca4, Ca5 and Ca7 with the same peptide fragment so that its multiple appearances would probably be due to the contamination. Similarly, 40 S ribosomal S5 (23 kDa) appeared in gel bands of Ca3, Ca4, Ca5, and Ca6 identified with the same peptide fragment (R)VNQAIVLLTTGAR(E) (Fig. 3). The molecular mass of Ca3 is close to 23 KDa, and the spectral percentage of Ca3 was highest among the 4 bands so that 40 S ribosomal S5 should be mainly associated with Ca3 and its presence in other 3 bands was probably due to contamination. 40 S ribosomal S5 was also identified in Cw6 with a different peptide fragment so that the relevant polypeptide identified in Cw6 might be a degraded product of 40 S ribosomal S5. Another argument for this hypothesis was that the TIC of the peptide fragment in Cw6 was only about 1–8% of those in CA3, Ca4, Ca5 and Ca6 (Supplemental Fig. 2). Similarly, the multiple appearances of late embryogenesis abundant protein D-19 in 6 CSPa bands and 3 CSPw bands seemed mainly due to the contamination (Fig. 4). The intact protein seemed like with Ca7 band as the protein molecular mass is 11 kDa and 3 peptide fragments were detected in the band digestion. The same peptide fragment KQQLGTEGYQEMGR appeared in Ca1, Ca3, Cw7, Cw8, and Cw9. The peptide fragment in Ca4, and CA 6 was identical, and extended the KQQLGTEGYQEMGR sequence further down with KGGLSNSDMSGGER. As the molecular mass of all these bands was greater than that of Ca7 with the same core peptides, it was justified to assume that the presence of the late embryogenesis abundant protein D-19 in Ca1, Ca3, Ca4, Cw7, Cw8 and Cw9 was attributed to contamination. The absence of the upper N-terminal fragment of Ca7 in these six contaminations could be due to the lower abundances (so that lower detection) of the protein in the six contaminated bands. The protein in Ca5 could also be a contamination. However, unlike in other gel bands, a C-terminal fragment was detected in addition to the core KQQLGTEGYQEMGR sequence. The additional C-terminal fragment might be a hint that the polypeptide of the protein in Ca5 was not exactly the same as others. In other words, it could exclude the possibility that it was altered somehow per mechanism 2 and/or 3. In the future, more rigorous work should be done to distinguish the effect of the real contamination concern from the true polypeptide fractions in gel separation.

**Figure 5:**
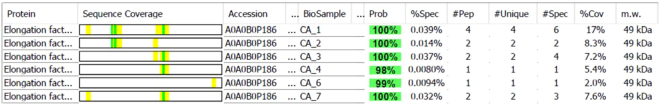
Sequence coverage of Elongation factor 1-alpha (A0A0B0P186, G. arboreum) by peptide fragments in CSPa.

On the other hand, multiple identification of elongation factor 1-alpha (49 kDa) seemed due to the degraded products of this protein instead of contamination. The protein was identified in Ca1 band (49 kDa) with 4 peptide fragments covered 74 of 447 amino acids of the protein (17% coverage) (Fig. 5). The peptide fragments identified in Ca2, Ca3, Ca4 and Ca7 did not overlap much, except for part of the peptide fragments of Ca1. Thus, the polypeptides in the four SDS-PAGE bands should be the degraded products of elongation factor 1-alpha in Ca1 gel band. The peptide fragment in Ca6 did not match any fragments in other bands and it was the highest TIC abundance (Supplemental Fig. 2). This observation suggested that the peptide fragment in Ca6 was not part of the polypeptide in Ca1 or other gel bands, rather complementary to each other as the whole elongation factor 1-alpha polypeptide or the C-terminal polypeptide related to Ca6 was excised by post-translational modification^37^. Oleosin 16.4 kDa was identified in Ca6 with four peptide fragments. The polypeptides of this protein were identified in lower molecular mass gel bands Ca7, Cw8, Cw9 and Cw10 with one peptide fragment which was also part of the peptides in Ca7, suggesting that the relevant polypeptides in these four gel bands were the degraded products of oleosin polypeptides in Ca7. Appearance of the degraded products in CSPw bands suggested the degradation occurred prior to the separation of the two protein fraction CSPw and CSPa. Indeed, oleosin in cottonseed is alkaline and hydrophobic proteins having three domains including amphipathic N and C termini and a central hydrophobic domain^26^. The degraded products of oleosin in Cw8, Cw9 and Cw10 were not in regions of the three hydrophobic domains, which might explain their appearance in the water soluble cottonseed protein fraction CSPw.

In addition, the molecular mass of some proteins identified was greater than that in the gel image. This could be also due to protein degradation. DNA polymerase alpha catalytic subunit-like protein (172 Kda) appeared in four gel bands (Ca4, Cw1, Cw2 and Cw4). However, their peptide fragments were all found in the late half C-terminal parts, a sign of peptide fraction of the whole protein. The same fragment of (K)CSVCHMDEEYENNLFLQCDKCR(M) of histone-lysine N-methyltransferase ATX2-like protein (123 kDa) detected in Ca1, Ca7 and Cw1 suggested degraded peptides of this protein was in the CSPa and CSPw protein products.

**Figure 6:**
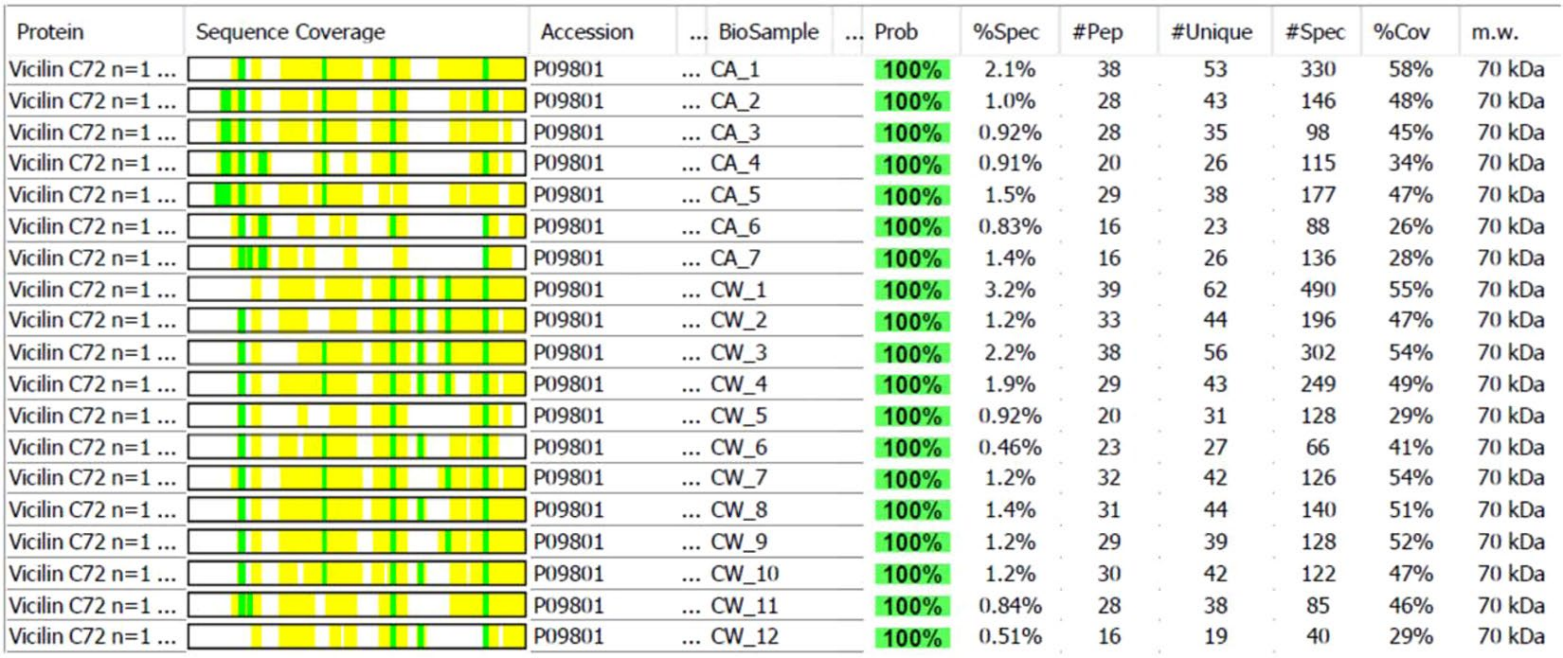
Sequence coverage of Vicilin C72 (P09801, G. hirsutum) by peptide fragments in CSPw and CSPa.

For those high-abundant proteins, it is difficult to clearly distinguish the mechanisms of their multiple appearances in multiple gel bands. It is likely that 2 or 3 mechanisms simultaneously contributed to the repeated appearance of the abundant proteins in multiple gel bands. Even though, careful examination of the features of the sequence coverages of these peptide fragments could still give us some insight into the cottonseed protein profiles. For example, analysis of the peptide fragments of vicilin C72 (G. hirsutum) showed that the fragments found in all 12 CSPw gel bands were consistently shorter than those in the 7 CSPa gel bands shown by the sequence coverage in the second column of Fig. 6. Sequence data comparison, represented by Ca5 and Cw5 in Supplemental Fig. 3, revealed that it is about 90 N-terminal amino acid (AA) fragment missed in CSPw bands. The short N-terminal sequences were observed in peptide fragments of vicilin GC72-A protein in 10 of 12 CSPw bands. Among them, the sequence coverage was 83 AAs short from the N-terminus in Cw9, 97 AAs short in Cw4, Cw10, and Cw11, 128 AAs short in Cw8, and 162 AAs short in Cw1, Cw2, Cw3, Cw6 and Cw12. These consistent observations implied that the distribution of the protein in all CSPa or CSPw bands of vicilin C72 might not be only due to the overwhelming amount (contamination) of the protein in cottonseed. Its CSPw version was probably a shortened fraction (or isoform) of the whole protein in CSPa. It would be interesting to determine if the characteristic was partly contributed to the lower hydrophobicity of CSPw than CSPa^29^. Isoforms of the same protein accession of seed storage protein have been reported in pea, soybean, and rapeseed^38–40^. In their 2-D SDS-PAGE image, Hu *et al*.^27^ reported that, out of 155 identified spots, 19 spots identified as vicilin A, 5 as vicilin B, 83 as legumin A, 27 as legumin B (27 spots), and 6 as vicilin-like protein. By mapping the peptides derived from MS analysis to the full-length protein sequences of vicilin A and B, Hu *et al*.^27^ found that isoforms of vicilin A (49, 35, 11, and 17 kDa) and vicilin B (49, and 17 kDa) were derived from the 70 kDa vicilin A and vicilin B prepropolypeptides through the cleavage of signal peptides together with the N-terminal fragments, respectively. Hu *et al*.^27^ further pointed out that protein modifications (e.g., glycosylation, phosphorylation, acetylation, and methylation) also likely contributed to the formation of these vicilin isoforms. The multiple peptides data of these proteins in Table 1 would be useful to investigate these post-translational modifications of cottonseed proteins.

As with vicilins, legumin isoforms may be formed through a series of modifications, including proteolytic cleavage and peptide degradation^27^. Isoform analysis through peptide mapping indicated that the 30-kDa polypeptide of legumin A derived from the C-terminal fragment of the 58 kDa prepropolypeptide. Legumin isoforms are commonly distributed at molecular mass of 30 kDa, 17–20 kDa, and 11–12 kDa as legumin A and at a molecular weight of 11–13 kDa as legumin B^27^. Like vicilin C72, it seemed that there were two groups of sequence coverage: one group about 40 amino acid longer N-terminal fragments than the other group. However, unlike vicilin 72, the two groups were not separated exclusively into CSPw and CSPa fractions, rather mixing in the two fractions. Thus, the two types of polypeptides (or isoforms) of legumin A might possess other features rather than the hydrophobicity. It was difficult by examination of the sequence coverage of legumin A to identify the isoforms of legumin A in these molecular mass regions although some differences in peptide sequences could be observed between the gel bands. On the other hand, the sequence coverage of the legumin B fragments in Ca4, Ca5, Cw1 and Cw12 were shorter than those in other gel bands. Thus, the peptides fragments of legumin B in these four gel bands might be related isoforms. In addition, there were minor differences in the peptide sequences between the fragments identified from the remaining 15 gel bands with the identical N- and C-terminal fragments. For example, a peptide sequence DNLLAQAFGDTR were not detected only in three (Ca1, Cw2, and Cw6) of the 15 gel bands. Further exploration of the multiple and different sequence coverage of these cottonseed proteins may shed more light on the evolution, post-translational modification and isoform of cottonseed and other oilseed proteins^25,27,41^. Further in-depth study could also provide novel insight into the functional utilization of the relevant peptide fragments to determine whether they are the peptide precursors or the degraded products^42–44^.

## Materials and Methods

### Cottonseed meal and protein extraction

Mill-scale produced defatted cottonseed meal was provided by Cotton, Inc. (Cary, NC, USA) and was used as the starting material for protein isolation as reported in He *et al*.^32,33^. Briefly, the water (CSPw)- and alkali-(CSPa) soluble protein fractions in the defatted cottonseed meal were sequentially extracted by water and 0.015 M NaOH, and then precipitated at pH 4.0 and 7.0, respectively. Both fractions were freeze-dried and kept in a desiccator at room temperature (22 °C) until use. The two protein isolates (i. e., CSPa and CSPw) were the products of the work^32^. The chemical analysis of the raw material and products are listed in Table 2.

**Table 2:** Chemical composition of defatted cottonseed meal (CSM) and its water-soluble (CSPw) and alkali-soluble (CSPa) protein fractions. Data compiled per He *et al*.^32^.

	Moisture	Ash	Protein	Oil	Crude Fiber	Cellulose	Hemicellulose	P	Ca	K	Mg	Na	S
	% of sample weight												
CSM	8.4	7.2	34.1	2.5	11.7	13.6	2.2	1.5	0.3	1.8	0.7	0.2	0.5
CSPw	8.9	4.6	64.4	3.4	0.9	1.6	0.0	1.1	0.1	0.9	0.2	0.1	0.8
CSPa	8.2	1.3	101.0	0.1	0.1	0.3	0.0	0.3	0.1	0.2	0.1	0.2	0.6

### Gel electrophoresis

CSPw and CSPa were dissolved in 20 mM NaOH at concentration of approximately 5 mg ml^−1^. Total proteins extracted in the supernatant were estimated by Coomassie Protein Assay Reagent (ThermoScientific) and separated by sodium dodecyl sulfate polyacrylamide gel (SDS-PAGE) using 4–12% Bis-Tris gel and MES running buffer (Invitrogen)^32^. Distinctive and prominent bands after Coomassie staining were excised from the gel, which were designated as Cw1–Cw12 for the CSPw sample and Ca1–Ca7 for the CSPa sample.

### MS analysis

Individual bands excised from the multiple SDS-PAGE lanes were pooled and subjected to in-gel trypsin digestion. The fragments in the digestions were analyzed by liquid chromatography-electrospray ionization-tandem spectrometry (LC-ESI-MS/MS). The mass spectral analysis was performed by UAB Mass Spectrometry/Proteomics Shared Facility (University of Alabama at Birmingham, Birmingham, Alabama, USA). The data were acquired with Bruker UltraFlex III MALDI ToF/ToF. The tandem mass spectral data generated were processed with SEQUEST and searched by Mascot against protein databases. The quantitative values of the normalized total ion current (TIC) of the peptide MS fragments were used as a relative measurement of the peptide abundance in the gel bands. Scaffold (version Scaffold_4.0.5, Proteome Software Inc., Portland, OR) was used to validate MS/MS based peptide and protein identifications^45^.

### Experimental design and statistical analysis

As reported in the work^32^, CSPw and CSPa were the co-products/byproducts of the pilot-scale production of washed cottonseed meal. The pilot testing was performed in triplicates. The mass yield and protein recovery were 0.7 ± 0.1% and 1.3 ± 0.1% for CSPw, 3.8 ± 0.4% and 11.8 ± 1.3% for CSPa, respectively. Both the yield and recovery data between the two products were statistically significantly different at α < 0.05.

For MS data treatments, peptide identifications were accepted if they could be established at greater than 80.0% probability by the Peptide Prophet algorithm^45^ with Scaffold delta-mass correction. Protein identifications were accepted if they could be established at greater than 99.0% probability and contained at least 2 identified peptides. Protein probabilities were assigned by the Protein Prophet algorithm^46^. Proteins that contained similar peptides and could not be differentiated based on MS/MS analysis alone were grouped to satisfy the principles of parsimony.

## Electronic supplementary material

Supplementary Information
