# Supplementary material for: Protein profiling of water and alkali soluble cottonseed protein isolates
Source: Sci Rep. 2018 Jun 18;8:9306. doi: 10.1038/s41598-018-27671-z (PMC6006339; doi:10.1038/s41598-018-27671-z)
Supplement: Supplementary file 1 — Supplementary Information [file 41598_2018_27671_MOESM1_ESM.pdf]

Supplementary information

Protein profiling of water and alkali soluble cottonseed protein isolates

Zhongqi He [1, *], Dunhua Zhang [2], and Heping Cao [1]

[1] USDA-ARS, Southern Regional Research Center, New Orleans, Louisiana, USA

[2] USDA-ARS, Aquatic Animal Health Research Unit, Auburn, Alabama, USA

* Corresponding author

Email address: zhongqi.he@ars.usda.gov (ZH)

Supplemental Figure 1. Taxonomy, protein identification and accession number of the polypeptides identified in CSPw and CSPa

Identified Proteins (70)	Taxonomy	RepID	Accession Number
(3R)-hydroxymyristoyl-[acyl-carrier-protein] dehydratase	Gossypium arboreum	A0A0B0MEY6_GOSAR	A0A0B0MEY6
1-acylglycerophosphocholine O-acyltransferase 1	Gossypium arboreum	A0A0B0N8P7_GOSAR	A0A0B0N8P7
2S albumin storage protein	Gossypium hirsutum	Q39787_GOSHI	Q39787
40S ribosomal S3-3-like protein	Gossypium arboreum	A0A0B0MK71_GOSAR	A0A0B0MK71 (+1)
40S ribosomal S5	Gossypium arboreum	A0A0B0PCC1_GOSAR	A0A0B0PCC1
60S ribosomal L23a	Gossypium arboreum	A0A0B0MZ40_GOSAR	A0A0B0MZ40
60S ribosomal protein L14-2	Gossypium arboreum	A0A0B0NJS5_GOSAR	A0A0B0NJS5
Adenosylhomocysteinase	Gossypium arboreum	A0A0B0NW28_GOSAR	A0A0B0NW28 (+1)
ADP,ATP carrier 1, chloroplastic-like protein	Gossypium arboreum	A0A0B0NBX6_GOSAR	A0A0B0NBX6
ALBINO3-like protein 1, chloroplastic	Gossypium arboreum	A0A0B0MQ65_GOSAR	A0A0B0MQ65
ATP synthase subunit beta	Gossypium hirsutum	C6GFP3_GOSHI	C6GFP3
Beta-xylosidase/alpha-L-arabinofuranosidase 2	Gossypium arboreum	A0A0B0NEY9_GOSAR	A0A0B0NEY9
Calcineurin subunit B	Gossypium arboreum	A0A0B0NRG9_GOSAR	A0A0B0NRG9
CBL-interacting serine/threonine-protein kinase 23	Gossypium arboreum	A0A0B0NVJ7_GOSAR	A0A0B0NVJ7 (+1)
Cellular nucleic acid-binding	Gossypium arboreum	A0A0B0MSB9_GOSAR	A0A0B0MSB9
Chaperone DnaJ	Gossypium arboreum	A0A0B0PLC6_GOSAR	A0A0B0PLC6
Chaperonin CPN60-2, mitochondrial	Gossypium arboreum	A0A0B0NFA8_GOSAR	A0A0B0NFA8 (+1)
Desiccation-related PCC3-06	Gossypium arboreum	A0A0B0N9Z5_GOSAR	A0A0B0N9Z5
DNA polymerase alpha catalytic subunit-like protein	Gossypium arboreum	A0A0B0NEL3_GOSAR	A0A0B0NEL3
Elongation factor 1-alpha	Gossypium arboreum	A0A0B0P186_GOSAR	A0A0B0P186
Eukaryotic initiation factor 4A-14	Gossypium arboreum	A0A0B0MUP2_GOSAR	A0A0B0MUP2
Exocyst complex component 7	Gossypium arboreum	A0A0B0PW69_GOSAR	A0A0B0PW69
Flagellar attachment zone 1	Gossypium arboreum	A0A0B0NTH5_GOSAR	A0A0B0NTH5
Fructose-bisphosphate aldolase, cytoplasmic isozyme	Gossypium arboreum	A0A0B0N935_GOSAR	A0A0B0N935
Glucose-6-phosphate isomerase, cytosolic	Gossypium arboreum	A0A0B0MUW2_GOSAR	A0A0B0MUW2 (+1)
Glyceraldehyde-3-phosphate dehydrogenase-2C cytosolic	Gossypium arboreum	A0A0B0MV73_GOSAR	A0A0B0MV73
Golgi to ER traffic 4	Gossypium arboreum	A0A0B0NB99_GOSAR	A0A0B0NB99
Guanylate-binding 5	Gossypium arboreum	A0A0B0PS63_GOSAR	A0A0B0PS63
HEAT repeat-containing 7A	Gossypium arboreum	A0A0B0Q1K1_GOSAR	A0A0B0Q1K1
Heat shock protein 70	Gossypium hirsutum	D2D320_GOSHI	D2D320
Histone H2B	Gossypium arboreum	A0A0B0NGQ0_GOSAR	A0A0B0NGQ0 (+1)
Histone-lysine N-methyltransferase ATX2-like protein	Gossypium arboreum	A0A0B0NJ00_GOSAR	A0A0B0NJ00
Isocitrate dehydrogenase [NADP]	Gossypium arboreum	A0A0B0MIG9_GOSAR	A0A0B0MIG9 (+1)
Kinase PVPK-1	Gossypium arboreum	A0A0B0NGF3_GOSAR	A0A0B0NGF3
Late embryogenesis abundant protein	Gossypium arboreum	A0A0B0P335_GOSAR	A0A0B0P335 (+1)
Late embryogenesis abundant protein D-19	Gossypium hirsutum	LE19_GOSHI	P09443
Legumin A	Gossypium hirsutum	LEGA_GOSHI	P09802
Legumin B	Gossypium hirsutum	LEGB_GOSHI	P09800
Lipid A export ATP-binding/permease MsbA	Gossypium arboreum	A0A0B0NQZ1_GOSAR	A0A0B0NQZ1
Low molecular weight heat shock protein	Gossypium hirsutum	A8WCV3_GOSHI	A8WCV3
Malate dehydrogenase-2C cytoplasmic	Gossypium arboreum	A0A0B0NLV9_GOSAR	A0A0B0NLV9
Mitochondrial 2-oxoglutarate/malate carrier	Gossypium arboreum	A0A0B0PR02_GOSAR	A0A0B0PR02
Oleosin 16.4 kDa	Gossypium hirsutum	OLEO7_GOSHI	P29528
Origin recognition complex subunit 1	Gossypium arboreum	A0A0B0MH05_GOSAR	A0A0B0MH05
Peptidyl-prolyl cis-trans isomerase cyp8	Gossypium arboreum	A0A0B0P0B2_GOSAR	A0A0B0P0B2
Phosphoglycerate kinase, cytosolic	Gossypium	A0A0B0NN51_GOSAR	A0A0B0NN51
Phototropin-1-like protein	Gossypium arboreum	A0A0B0MNY7_GOSAR	A0A0B0MNY7
Proteasome subunit beta type-6-like protein	Gossypium arboreum	A0A0B0PP74_GOSAR	A0A0B0PP74
Protein argonaute 4-like protein	Gossypium arboreum	A0A0B0NX11_GOSAR	A0A0B0NX11
Protein lin-54	Gossypium arboreum	A0A0B0P3S6_GOSAR	A0A0B0P3S6
Protein neuralized	Gossypium arboreum	A0A0B0PWY7_GOSAR	A0A0B0PWY7
Protein NLP8-like protein	Gossypium arboreum	A0A0B0MLL9_GOSAR	A0A0B0MLL9
Protein yeeZ	Gossypium arboreum	A0A0B0MFB1_GOSAR	A0A0B0MFB1
Putative ATP-dependent RNA helicase DHX36	Gossypium arboreum	A0A0B0PXH7_GOSAR	A0A0B0PXH7
Putative vacuolar sorting-associated protein 13B	Gossypium arboreum	A0A0B0NXF5_GOSAR	A0A0B0NXF5
Putative zinc finger CONSTANS-LIKE 11-like protein	Gossypium arboreum	A0A0B0Q151_GOSAR	A0A0B0Q151
Replication A 70 kDa DNA-binding subunit	Gossypium arboreum	A0A0B0MSM1_GOSAR	A0A0B0MSM1
Rhamnogalacturonate lyase	Gossypium arboreum	A0A0B0M5N8_GOSAR	A0A0B0M5N8
RING finger and CHY zinc finger domain-containing 1	Gossypium arboreum	A0A0B0NK68_GOSAR	A0A0B0NK68
Serine/threonine-protein kinase SIK3	Gossypium arboreum	A0A0B0NNV2_GOSAR	A0A0B0NNV2
Transcription factor BIM2-like protein	Gossypium arboreum	A0A0B0PND5_GOSAR	A0A0B0PND5
Transcription initiation factor TFIID subunit 1-B-like protein	Gossypium arboreum	A0A0B0NUG8_GOSAR	A0A0B0NUG8
Vacuolar-sorting receptor 1-like protein	Gossypium arboreum	A0A0B0PFQ0_GOSAR	A0A0B0PFQ0
Vicilin C72	Gossypium hirsutum	VCLB_GOSHI	P09801
Vicilin C72	Gossypium arboreum	A0A0B0N1K7_GOSAR	A0A0B0N1K7
Vicilin GC72-A	Gossypium hirsutum	VCLA_GOSHI	P09799
Vicilin-like antimicrobial peptides 2-1	Gossypium arboreum	A0A0B0PET1_GOSAR	A0A0B0PET1
Vicilin-like antimicrobial peptides 2-2	Gossypium arboreum	A0A0B0PUP3_GOSAR	A0A0B0PUP3
V-type proton ATPase subunit B 1	Gossypium arboreum	A0A0B0PNB1_GOSAR	A0A0B0PNB1 (+1)
Zfy1	Gossypium arboreum	A0A0B0NWH3_GOSAR	A0A0B0NWH3

Supplemental Figure 2. Quantitative values (normalized total ion current) of the polypeptides identified in CSPw and CSPa

	CSPa							CSPw											
Identified Proteins (70)	1	2	3	4	5	6	7	1	2	3	4	5	6	7	8	9	10	11	12
Vicilin C72	9.1E+08	2.0E+08	1.2E+08	1.2E+08	4.7E+08	4.4E+08	7.7E+08	9.2E+08	1.4E+08	5.5E+08	6.9E+08	4.6E+08	7.1E+07	2.2E+08	6.7E+08	4.9E+08	3.7E+08	1.6E+08	1.4E+08
Legumin A	1.8E+06	3.0E+07	1.1E+09	8.8E+08	4.4E+08	5.3E+08	3.3E+08	2.4E+06	2.2E+08	1.3E+08	6.8E+07	5.5E+08	1.4E+09	1.4E+08	1.6E+08	3.0E+08	6.7E+08	1.3E+09	3.0E+08
Vicilin GC72-A	1.2E+08	1.5E+09	4.1E+08	6.2E+07	1.6E+08	4.0E+08	3.8E+07	1.5E+08	1.4E+09	6.3E+08	5.1E+08	3.7E+08	1.0E+08	4.6E+08	5.7E+08	4.5E+08	4.1E+08	1.1E+08	9.8E+07
Legumin B	7.7E+06	1.2E+07	1.1E+08	7.5E+08	4.0E+08	9.0E+07	2.3E+07	8.8E+06	8.6E+06	4.9E+07	1.1E+08	8.6E+07	3.3E+08	9.0E+08	5.1E+08	2.3E+08	1.8E+08	1.4E+08	1.4E+09
Vicilin-like antimicrobial p	1.8E+05	3.5E+06	3.3E+07	8.7E+04	1.5E+06	2.0E+06	7.5E+06	1.2E+06	7.2E+05	1.0E+07	3.7E+05	4.2E+06	3.6E+04	2.0E+06	1.3E+07	7.2E+05	8.3E+05	8.7E+07	0
Vicilin C72 n=1	8.9E+08	1.9E+08	1.2E+08	1.1E+08	4.6E+08	4.4E+08	7.5E+08	8.5E+08	1.3E+08	5.5E+08	5.4E+08	4.6E+08	7.0E+07	2.1E+08	0.0E+00	4.5E+08	3.0E+08	1.6E+08	0
Late embryogenesis abun	21893	0	1817500	377920	674620	1139400	1413700	0	0	0	0	0	0	98353	381440	93072	0	0	0
2S albumin storage protei	0	18491	265360	75364	65950	0	366950	0	0	15741	16607	0	0	0	496160	0	0	0	2412700
Flagellar attachment zone	0	0	0	11993	0	0	314370	0	0	0	0	0	0	0	0	1003000	0	0	0
ATP synthase subunit beta	31060	37299	297300	15715	296580	2347800	457650	0	0	0	0	0	0	0	0	0	0	0	0
Elongation factor 1-alpha	523660	58919	1229800	25211	0	1966700	334420	0	0	0	0	0	0	0	0	0	0	0	0
Heat shock protein 70	0	16781	1202600	0	796510	0	283440	0	0	0	4749.6	0	0	0	0	0	0	0	0
Vicilin-like antimicrobial p	0	0	1313100	0	116840	0	0	0	0	0	74167	0	360790	24506	0	546440	55430	0	0
Transcription initiation fac	119670	0	0	0	0	0	0	0	640120	0	0	65228	0	0	0	208650	253740	0	0
DNA polymerase alpha ca	0	0	0	15031	0	0	0	332.45	7569.3	0	196430	0	0	0	0	0	0	0	0
Protein lin-54	1415	0	0	0	0	231960	0	20602	12692	40460	0	0	0	0	0	93947	0	0	0
Glyceraldehyde-3-phosph	0	0	1597700	3580800	368730	4573100	468130	0	0	0	0	0	0	0	0	0	0	0	0
40S ribosomal S5	0	0	185650	74188	737830	253830	0	0	0	0	0	0	5748.3	0	0	0	0	0	0
Oleosin 16.4 kDa	0	0	0	0	0	6958000	735630	0	0	0	0	0	0	0	82234	265430	304300	0	0
Late embryogenesis abun	0	109520	2685400	37081	121190	0	0	0	0	0	0	0	0	0	0	0	0	0	0
Malate dehydrogenase-2(	19582	0	548290	125560	235080	76759	0	0	0	0	0	0	0	0	0	0	0	0	0
Eukaryotic initiation facto	26333	31553	854470	0	0	215400	95559	0	0	0	0	0	0	0	0	0	0	0	0
ALBINO3-like protein 1, ch	0	0	0	9816.4	263540	0	349730	0	0	0	0	0	0	0	0	0	0	0	0
RING finger and CHY zinc f	939.72	11344	0	0	0	0	0	0	3483.7	0	0	0	0	0	203470	0	0	0	0
Histone H2B	0	0	2147300	0	0	373250	145920	0	0	0	0	0	0	0	0	0	0	0	0
Putative ATP-dependent R	1017.2	131510	0	6547.7	0	0	0	0	0	0	0	20663	54240	0	0	0	0	0	0
Desiccation-related PCC3-	0	0	776370	155450	153780	0	0	0	0	0	0	0	0	0	0	0	0	0	0
Histone-lysine N-methyltr	5515.2	0	0	0	0	0	197020	877.07	0	0	0	0	0	0	0	0	0	0	0
Cellular nucleic acid-bindi	0	0	0	0	0	0	104460	3866.8	0	0	0	0	0	0	0	0	0	48416	0
Proteasome subunit beta	0	0	70193	335440	0	0	0	0	0	0	0	0	0	0	0	0	0	0	0
Rhamnogalacturonate lya	0	0	0	0	0	0	0	0	0	0	0	0	0	0	0	8937900	0	0	0
Protein NLP8-like protein	0	3366.8	0	0	42600	0	0	0	0	0	0	0	0	0	0	0	0	0	0
Protein neuralized	0	35975	0	0	0	267190	0	0	0	0	0	0	0	5031.3	0	0	0	0	0
60S ribosomal protein L14	0	0	0	0	62591	907580	0	0	0	0	0	0	0	0	0	371480	0	0	0
Replication A 70 kDa DNA	0	101110	0	0	0	0	0	0	6795.7	0	24271	0	0	0	0	0	0	0	0
Low molecular weight hea	0	0	0	105080	1056000	0	199060	0	0	0	0	0	0	0	0	0	0	0	0

Phosphoglycerate kinase,	186990	549560	0	0	0	0	0	0	0	0	0	0	0	0	0	0	0	0	0
Glucose-6-phosphate ison	0	0	173430	0	12045	0	0	9705.4	15150	0	0	0	0	0	0	0	0	0	0
Mitochondrial 2-oxoglutai	0	62370	0	0	0	0	0	0	16541	0	0	0	0	0	0	516030	0	0	0
Peptidyl-prolyl cis-trans is	0	78012	0	0	0	0	608580	0	0	0	0	0	0	0	0	0	654080	0	0
Adenosylhomocysteinase	0	106870	0	0	0	0	0	0	0	0	0	0	0	0	0	193710	70178	0	0
Protein argonaute 4-like p	0	0	0	0	0	0	58379	7714.6	0	0	14588	0	0	0	0	0	0	0	0
Vacuolar-sorting receptor	0	0	0	0	0	0	0	23956	0	0	0	0	0	0	0	0	0	0	0
60S ribosomal L23a	0	0	0	311450	391970	0	0	0	0	0	0	0	0	0	0	0	0	0	0
Isocitrate dehydrogenase	0	414650	0	0	0	0	0	0	0	0	0	0	0	0	0	0	0	0	0
Zfy1	0	0	0	0	46743	0	0	0	0	0	0	0	0	69180	108060	0	0	0	0
CBL-interacting serine/thr	0	0	0	0	0	311490	0	0	0	0	0	0	0	20561	0	0	0	0	0
Putative vacuolar sorting-	0	0	0	0	0	0	0	3550.2	170810	0	0	0	0	0	0	0	0	0	0
Kinase PVPK-1	0	0	0	0	0	0	0	113060	0	0	0	0	0	0	0	0	0	0	0
HEAT repeat-containing 7	0	0	0	0	0	0	0	0	7950.8	12023	0	0	0	0	0	0	0	0	0
Putative zinc finger CONS	0	13353	0	0	0	0	0	399.9	0	0	0	0	0	0	0	0	0	0	0
Origin recognition comple	0	0	0	0	0	0	0	0	0	0	69865	0	0	0	0	0	0	0	0
Chaperonin CPN60-2, mit	0	0	507080	0	0	0	0	0	0	0	0	0	0	49862	0	0	0	0	0
V-type proton ATPase sub	0	0	0	0	0	795600	0	0	0	0	0	0	0	0	0	0	0	0	0
Fructose-bisphosphate ald	3752.2	83724	0	0	0	0	0	0	0	0	0	0	0	0	0	0	0	0	0
Beta-xylosidase/alpha-L-a	0	0	0	0	24352	0	0	0	0	0	0	0	0	0	0	0	0	77166	0
Lipid A export ATP-bindin	0	0	0	0	27372	0	0	0	11835	0	0	0	0	0	0	0	0	0	0
Transcription factor BIM2	0	0	0	0	0	290450	0	6788.4	0	0	0	0	0	0	0	0	0	0	0
1-acylglycerophosphocho	0	0	190280	0	0	0	0	0	0	0	0	0	0	0	0	0	0	0	0
Protein yeeZ	0	0	0	0	0	0	0	0	0	0	0	21864	0	0	0	0	0	0	0
(3R)-hydroxymyristoyl-[ac	0	0	0	0	408130	0	0	0	0	0	0	0	0	0	0	0	0	0	0
Guanylate-binding 5	0	0	0	0	0	0	0	0	0	0	0	10251	0	0	0	0	0	0	0
Calcineurin subunit B	0	0	0	0	0	0	0	1859500	0	0	0	0	0	0	0	0	0	0	0
Phototropin-1-like protei	26238	0	0	0	0	0	0	0	0	0	0	0	0	0	0	0	0	0	0
Serine/threonine-protein	0	0	0	0	0	0	0	42932	0	0	0	0	0	0	0	0	0	0	0
ADP,ATP carrier 1, chloro	0	0	0	0	0	0	0	0	5829.2	0	0	0	0	0	0	0	0	0	0
Golgi to ER traffic 4	0	0	0	0	0	0	0	0	0	0	0	0	0	0	0	221620	0	0	0
40S ribosomal S3-3-like p	0	0	250480	0	0	0	0	0	0	0	0	0	0	0	0	0	0	0	0
Exocyst complex compon	0	0	0	0	0	0	0	0	16027	0	0	0	0	0	0	0	0	0	0
Chaperone DnaJ	0	0	0	0	0	0	0	0	2695.8	0	0	0	0	0	0	0	0	0	0

Supplemental Figure 3. The whole amino acid sequence of Vicilin GC72-A and its peptide fragments (colored) identified in Ca5 and Cw5 gel bands

Ca5 gel band

MVRNKSACVV	LLFSLFLSFG	LLCSAKDFPG	RRGDDDPPKR	YEDCRR**RCEW**
**DTRGQKEQQQ**	**CEESCKSQYG**	**EKDQQQRHRP**	**EDPQRRYEEC**	**QQECRQQEER**
QRPQCQQRCL	K**RFEQEQQQS**	**QR**QFQECQQH	CHQQEQRPER	KQQCVRECRE
RYQENPWRR**E**	**REEEAEEEET**	**EEGEQEQSHN**	**PFHFHR**RSFQ	SR**FREEHGNF**
**R**VLQRFASRH	PILRGINEFR	**LSILEANPNT**	**FVLPHHCDAE**	**K**IYLVTNGR**G**
**TLTFLTHENK**	**ESYNVVPGVV**	**VRVPAGSTVY**	**LANQDNKEKL**	**IIAVLHR**PVN
NPRQFEEFFP	AGSQRPQSYL	RAFSREILEP	AFNTR**SEQLD**	**ELFGGR**QSHR
RQQGQGMFR**K**	**ASQEQIRALS**	**QEATSPR**EKS	GERFAFNLLY	RTPRYSNQNG
RFYEACPREF	RQLSDINVTV	SALQLNQGSI	FVPHYNSKAT	FVVLVNEGNG
YVEMVSPHLP	R**QSSFEEEEE**	**QQQEQEQEEE**	**RR**SGQYRKIR	SQLSR**GDIFV**
**VPANFPVTFV**	**ASQNQNLRMT**	**GFGLYNQNIN**	**PDHNQR**IFVA	GKINHVRQWD
SQAKELAFGV	SSR**LVDEIFN**	**NNPQESYFVS**	**R**QRQRASE	

Cw5 gel band

MVRNKSACVV	LLFSLFLSFG	LLCSAKDFPG	RRGDDDPPKR	YEDCRRRCEW
DTRGQKEQQQ	CEESCKSQYG	EKDQQQRHRP	EDPQRR**YEEC**	**QQECR**QQEER
QRPQCQQRCL	K**RFEQEQQQS**	**QR**QFQECQQH	CHQQEQRPER	KQQCVRECRE
RYQENPWRRE	REEEAEEEET	EEGEQEQSHN	PFHFHRRSFQ	SR**FREEHGNF**
**R**VLQRFASRH	PILRGINEFR	LSILEANPNT	FVLPHHCDAE	KIYLVTNGR**G**
**TLTFLTHENK**	**ESYNVVPGVV**	**VRVPAGSTVY**	**LANQDNKEK**L	IIAVLHRPVN
NPRQFEEFFP	AGSQRPQSYL	R**AFSREILEP**	**AFNTRSEQLD**	**ELFGGR**QSHR
**RQQGQGMFRK**	**ASQEQIRALS**	**QEATSPR**EKS	GERFAFNLLY	RTPRYSNQNG
RFYEACPREF	RQLSDINVTV	SALQLNQGSI	FVPHYNSKAT	FVVLVNEGNG
YVEMVSPHLP	RQSSFEEEEE	QQQEQEQEEE	RRSGQYRKIR	SQLSR**GDIFV**
**VPANFPVTFV**	**ASQNQNLRMT**	**GFGLYNQNIN**	**PDHNQR**IFVA	GKINHVRQWD
SQAK**ELAFGV**	**SSR**LVDEIFN	NNPQESYFVS	RQRQRASE	
